# Select Cases of the Disorder Commonly Termed the Paralysis of the Lower Extremities

**Published:** 1782

**Authors:** 


VI. *Select Cafes of the Diforder commonly termed the Paralyfis of the Lower Extremities. By* John Jebb, M. D. F. R. S. 8vo. Stockdale, *London*, 1782. 74 pages. 1 s.

THESE cafes, which are fifteen in number, being related with great accuracy, may be confidered as a valuable fupplement to the treatife lately publifhed on the fame fubject by Mr.

Mr. Pott. The greater number of them, it ſeems, occurred in St. Bartholomew's hoſpital, under the immediate management of that gentleman, and the relation of them will certainly tend to confirm his theory, and explain the reaſons of his practice. In his hiſtory of each caſe, our author has very properly confined himſelf ſolely to evident ſymptoms and the patient's narrative; being fully ſatisfied, that to deſcribe diſorders according to the forms in which they really evidence themſelves to the ſenſes, with a careful attention to the patient's feelings, is the moſt likely method of acquiring a knowledge of their cauſes and cure.

As it would be impoſſible to do juſtice to theſe caſes in an abridged view of them, we ſhall content ourſelves with giving the following general concluſions, which have been ſuggeſted to the ingenious author by thoſe now publiſhed, and above twice the number of others to which he has carefully attended:

" 1. That the cauſtics, which were indiſcri-

" minately applied in every ſtage of the diſeaſe,

" were the efficient means of cure in a majority

" of the caſes, and that they generally ſucceeded

" when the caſe could with propriety be termed

" a fair one.

" 2. That

" 2. That in the remainder, with one or two " exceptions, they produced an evident effect " in reftoring fenfibility and fome degree of " motion.

" 3. That in the unfuccefsful cafes, the pa- " tients died exhaufted by hectic fever, and the " genuine effects of the diftemper, and did not " appear to be prejudiced, in the remoteft degree, " by the application of the cauftics."

In two or three inftances our author has ob- ferved the upper cervical vertebræ affected. In one of thefe a collection of matter was found in the vicinity of the fecond vertebra. The un- happy fufferer, upon every motion of his head, felt a pain defcend in the courfe of the fpinal marrow, with general numbnefs, and fometimes pricking pains at the extremities of his toes and fingers.

From reflecting upon cafes of lumbar abfcefs, Dr. Jebb has been led to conclude, that, in thefe inftances of the diftempered fpine, where a pro- tuberance, evidencing a mechanical derangement of the parts, is connected with the paralyfis of the lower extremities, the purulent matter, gene- rated while the caries is advancing, is prevented from efcaping downwards by the thick, ligamen- tous fubftance, that covers the bodies of the vertebræ, vertebræ, and that this fluid thus detained aſſiſts in the further corroſion of thoſe parts. In other caſes, he obſerves, it may be ſuppoſed, that the matter formed by ulceration either originated on the outſide of the ligamentous covering of the ſpine, or elſe burſts from its confinement within that aponeuretic expanſion, and making its way in the courſe of the pſoæ muſcles, produces that peculiar form of the diſorder, to which the name of lumbar abſceſs is aſſigned. In ſuch circumſtances—he aſks—would it not be reaſonable to open an outlet for the collected fluid, as ſoon as the fluctuating tumour in the groin, and other ſymptoms, ſhall aſcertain the nature of the complaint, by means of a cauſtic applied to the moſt depending part; and at the ſame time to form large iſſues on each ſide of the ſpinous proceſſes of the firſt or ſecond vertebra of the loins?

Dr. Jebb is perſuaded, that if all the caſes of a diſtempered ſpine, which have occurred during the laſt five years at St. Bartholomew's hoſpital, were faithfully and circumſtantially reported, great advantage would be derived to medical knowledge, and the mode of treatment, recommended by Mr. Pott, be ſtill more evidently demonſtrated. In another part of his pamphlet he he describes the utility which would accrue to students in physic, were the physicians of our hospitals to point out to them such particular cases as seemed most likely to afford opportunities of improvement, and to draw up regular and well-digested histories of such cases as might appear most deserving of attention, and insert them, properly authenticated, with an account, where it could be procured, of the appearances on dissection, in the books of the hospital. Such histories and details—he emphatically remarks—"would be attended with public as "well as private advantage; they would be "analogous to the reports and year books of our "lawyers—to the recorded observations of the "appearances in the heavens—and might be "resorted to as authorities, and as evidences of "Nature's powers, and of Nature's laws."

In an appendix the author relates a case of catalepsy. The patient was a young lady, who had laboured under the disorder for nine months. It was accompanied with a variety of hysterical symptoms, and was evidently exasperated at the approach of the catamenia, which were constantly present at the regular period. When he first saw her, she was seized with the disorder as soon as his arrival was announced. "She was em-

"ployed—says he—in netting, and was passing

"the needle through the mesh; in which position

"she immediately became rigid, exhibiting, in

"a very pleasing form, a figure of death-like

"sleep, beyond the power of art to imitate, or

"the imagination to conceive. Her forehead

"was serene, her features perfectly composed.

"The paleness of her colour, her breathing at

"a distance being also scarce perceptible, ope-

"rated in rendering the similitude to marble

"more exact and striking. The position of her

"fingers, hands, and arms, was altered with

"difficulty; but preserved every form of flexure

"they acquired: nor were the muscles of the

"neck exempted from this law; her head

"maintaining every situation, in which the hand

"could place it, as firmly as her limbs.

"Upon gently raising the eyelids, they imme-

"diately closed, with a degree of spasm. The

"iris contracted upon the approach of a candle,

"as in a state of vigilance; the eye-ball itself

"was slightly agitated with a tremulous motion,

"not discernible when the eye-lid had descended.

"About half an hour after my arrival, the

"rigidity in her limbs and statue-like appearance

"being yet unaltered, she sung three plaintive

"songs, in a tone of voice so elegantly expressive,

"and with fuch affecting modulation, as evi-
"dently pointed out how much the moft power-
"ful paffion of the mind was concerned in the
"production of her diforder, as indeed her hif-
"tory confirmed. In a few minutes afterwards
"fhe fighed deeply, and the fpafm in her limbs
"was immediately relaxed. She complained
"that fhe could not open her eyes, her hands
"grew cold, a general tremor followed; but in
"a few feconds recovering intirely her recollec-
"tion and powers of motion, fhe entered into
"a detail of her fymptoms, and the hiftory of
"her complaints. . . . . . . . After fhe had dif-
"courfed for fome time with apparent calmnefs,
"the univerfal fpafm fuddenly returned. Her
"features now affumed a different form, de-
"noting a mind ftrongly impreffed with anxiety
"and apprehenfion. At times fhe uttered fhort
"and vehement exclamations, in a piercing tone
"of voice, expreffive of the paffions that agitated
"her mind; her hands being ftrongly locked in
"each other, and all her mufcles, thofe fubfer-
"vient to fpeech excepted, being affected with
"the fame rigidity as before."

She had no recollection whatever of what paffed in the fits, which returned once or twice a day, fometimes more frequently. Various remedies remedies were tried without much advance, but at length a plaſter of opium and camphor, applied to the pit of the ſtomach, was found to have a good effect.

Obſerving the utility of this application, and reflecting upon the many tokens of debility which her ſtomach exhibited, our author's attention was directed to the ſtrengthening of that organ, and notwithſtanding the diſcouraging circumſtances that had formerly attended the exhibition of the bark, determined to make another trial of its power.

He choſe the following form of preparation, which Dr. Whytt had found to be particularly ſerviceable in hyſterical complaints.

℞. Cort. Peruv. p. uncias duas,

Rad. gentian.

Cort. aurantior. āā drachmas ſex, miſce: infunde in ſpir. vinoſ. gallic. ℔ij. in balneo arenæ per dies ſex et cola.

Of this tincture the patient took two drachms (which was as much as her ſtomach would bear) twice a day, diluted with an ounce and a half of water, with the addition of a drachm of the compound ſpirit of lavender. Inſtead of the common kinds of tea, ſhe drank an infuſion of the outward rind of lemon, and occaſionally took

ſome drops of Hoffman's anodyne liquor, or of laudanum.

She perſiſted in this courſe with evident advantage. Her fits grew leſs frequent, returning faintly after a week or fortnight's interval: her ſpirits improved, her ſtrength increaſed, and at length, without the uſe of any other medicine, ſhe became entirely free from all complaint.

